# Supplementary figures and images for: Multinucleated Retinal Pigment Epithelial Cells Adapt to Vision and Exhibit Increased DNA Damage Response
Source: Cells. 2022 May 5;11(9):1552. doi: 10.3390/cells11091552 (PMC9103592; doi:10.3390/cells11091552)

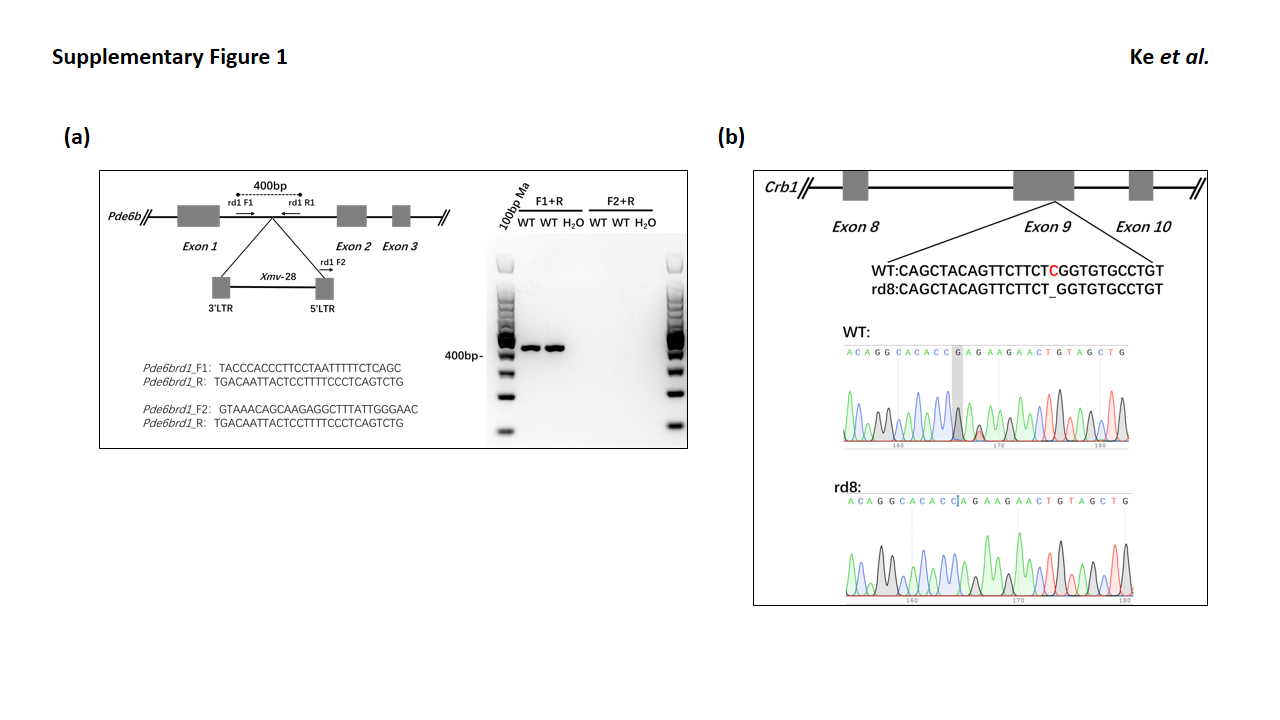

Supplement: Supplementary file 1 [file cells-11-01552-s001.zip › cells-1667961-supplementary.tif]
